# hacksig: a unified and tidy R framework to easily compute gene expression signature scores

**DOI:** 10.1093/bioinformatics/btac161

**Published:** 2022-03-18

**Authors:** Andrea Carenzo, Federico Pistore, Mara S Serafini, Deborah Lenoci, Armando G Licata, Loris De Cecco

**Affiliations:** Molecular Mechanisms Unit, Department of Research, Fondazione IRCCS Istituto Nazionale dei Tumori, Milan 20133, Italy; Head and Neck Cancer Medical Oncology 3 Department, Fondazione IRCCS Istituto Nazionale dei Tumori, Milan 20133, Italy; Molecular Mechanisms Unit, Department of Research, Fondazione IRCCS Istituto Nazionale dei Tumori, Milan 20133, Italy; Molecular Mechanisms Unit, Department of Research, Fondazione IRCCS Istituto Nazionale dei Tumori, Milan 20133, Italy; Molecular Mechanisms Unit, Department of Research, Fondazione IRCCS Istituto Nazionale dei Tumori, Milan 20133, Italy; Molecular Mechanisms Unit, Department of Research, Fondazione IRCCS Istituto Nazionale dei Tumori, Milan 20133, Italy

## Abstract

**Summary:**

Hundreds of gene expression signatures have been developed during the last two decades. However, due to the multitude of development procedures and sometimes a lack of explanation for their implementation, it can become challenging to apply the original method on custom data. Moreover, at present, there is no unified and tidy interface to compute signature scores with different single sample enrichment methods. For these reasons, we developed hacksig, an R package intended as a unified framework to obtain single sample scores with a tidy output as well as a collection of manually curated gene signatures and methods from cancer transcriptomics literature.

**Availability and implementation:**

The hacksig R package is freely available on CRAN (https://CRAN.R-project.org/package=hacksig) under the MIT license. The source code can be found on GitHub at https://github.com/Acare/hacksig.

**Supplementary information:**

Supplementary data are available at *Bioinformatics* online.

## 1 Introduction

A gene signature can be defined as a set of genes sharing a common pattern of expression in relation to a certain phenotype or biological process (Cantini *et al.*, 2018). In years, several enrichment methods have been developed for microarray and RNA-seq data in order to summarize the information coming from gene sets into a single score. This could lead to a more meaningful interpretation of results, both from a biological and clinical point of view, as well as a reduction in the effects of the curse of dimensionality problem affecting genomic studies (i.e. many more variables than samples).

Gene signature scoring methods can follow a number of approaches. For example, by averaging the expression values for genes in a signature (Rooney *et al.*, 2015); or also, by fitting a penalized regression model and then computing single sample scores as a weighted sum between fitted model coefficients and gene expression values (De Cecco *et al.*, 2014). However, it is not always straightforward to directly apply gene signature scoring methods from the literature to custom data. Sometimes details about how a method is implemented are vague and open to interpretation. Other times gene identifiers and eventual model coefficients must be extracted manually from .pdf files or even from images. So, computing signature scores with the original publication method can become a time-consuming procedure even in the best-case scenario.

Gene expression signature scores can be derived using either the original publication procedure or one of five single sample enrichment methods, all of which are collected into two distinct R packages: GSVA, implementing four methods and singscore, enabling to compute enrichment scores with the self-titled procedure (Hänzelmann *et al.*, 2013; Foroutan *et al.*, 2018). The interfaces of these R packages are obviously different and designed to work primarily within the Bioconductor ecosystem (Huber *et al.*, 2015). Hence, none of them do provide a tidy output as intended by Wickham *et al.* (2019) that is a consistent format for successive data analysis pipelines such as data visualization with ggplot2 and modeling with tidymodels.

Herein, we propose the R package hacksig in order to address the above-mentioned issues and hence to:

1. compute single sample scores for both custom and manually curated gene expression signatures either with the original publication method or with three alternatives, namely the combined z-score, single sample GSEA (ssGSEA) and singscore (Lee *et al.*, 2008; Barbie *et al.*, 2009; Foroutan *et al.*, 2018);
2. provide a unified, simple interface and a tidy output.

## 2 Features and methods

The current release of hacksig includes 23 cancer transcriptomics gene signatures, which were selected mainly due to our interest in the tumor microenvironment biology and its possible influence on response to treatment in head and neck cancer patients (Van den Bossche *et al.*, 2022). The function get_sig_info() can be used to retrieve IDs, associated keywords, DOIs for the original publication and a brief description for each signature (see also Supplementary Table S1).

Most of the package functions require a normalized gene expression matrix as a primary input argument, with genes as rows and samples as columns. Hence, both microarray and RNA-seq normalized data are supported. A list of official HUGO gene symbols composing each implemented signature can be obtained with get_sig_genes().

### 2.1 The main function

**Fig. 1.**
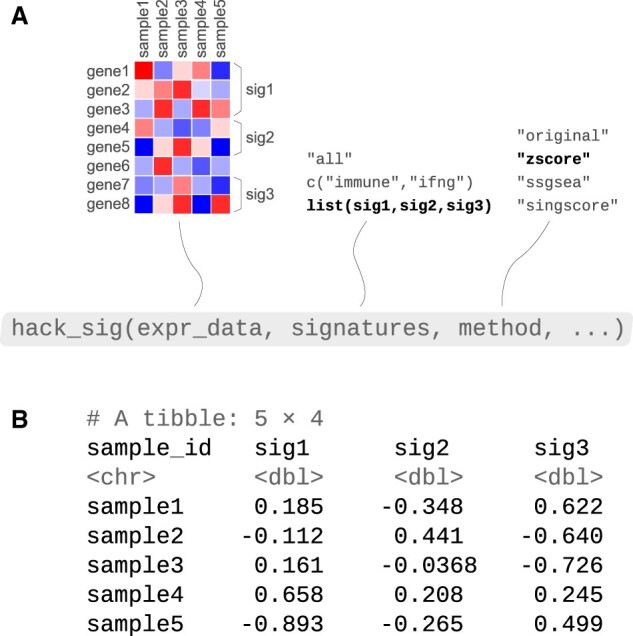
Syntax and output for the main function hack_sig(). (**A**) Possible choices for its three main arguments expr_data, signatures and method are shown. (**B**) An example output resulting from choosing arguments in bold is shown (i.e. a custom list of gene sets and the combined z-score method). The ellipsis represents additional arguments controlling options for the enrichment methods

The most important function of the package is hack_sig(), which can be used to easily compute gene expression signature scores in a number of ways. Figure 1A summarizes the syntax and different choices for arguments of the function.

If only an expression matrix is given in input, then hack_sig() will compute scores with the original procedure for all the implemented signatures, except those related to CINSARC (Chibon *et al.*, 2010), ESTIMATE (Yoshihara *et al.*, 2013) and Immunophenoscore (Charoentong *et al.*, 2017), for which dedicated functions exist (see next section). Anyway, signature scores can also be derived with one of the three possible single sample enrichment methods by setting the argument method to one of ‘zscore’, ‘ssgsea’ or ‘singscore’, corresponding to the combined z-score, ssGSEA and singscore, respectively. This will cause hack_sig() to compute enrichment scores with that particular procedure for all the implemented signatures. In addition, other optional arguments regarding single sample methods can be modified, such as the exponent in the running sum statistic of ssGSEA or its type of normalization.

It is possible to select just a particular group of signatures or to compute scores for a custom list of gene sets by means of the argument signatures, which is set to ‘all’ (i.e. all the implemented signatures) by default. If signatures is a character vector (e.g. c(‘immune’,‘ifng’)) with one or more valid keywords, hack_sig() will compute scores only for signatures matching those strings, either with the original procedure or with one of the three single sample alternatives depending on the choice of method. If signatures is a custom list of gene sets, then hack_sig() will compute scores with the procedure specified in the method argument, which cannot be set to ‘original’ in this case. If method is not specified, raw ssGSEA scores will be obtained by default for custom gene sets.

In general, the result of calling hack_sig() will be a tibble (i.e. a modern redefining of the data.frame R class) with one row per sample, a column indicating sample IDs and one column for each considered gene signature giving the corresponding scores (Fig. 1B).

### 2.2 Other features

Although hack_sig() can be used to compute scores for most of the gene signatures included in the package, there are three particular methods which for us deserve their own function implementation. These are hack_cinsarc(), which implements the CINSARC classification (Chibon *et al.*, 2010; Lesluyes and Chibon, 2020); hack_estimate(), which computes the immune, stroma, ESTIMATE and tumor purity scores as in Yoshihara *et al.* (2013); hack_immunophenoscore(), giving immune marker scores together with the Immunophenoscore (Charoentong *et al.*, 2017).

Before computing enrichment scores, it should be considered good practice to always check if genes composing a signature are well represented in the expression matrix. For this reason, we developed check_sig(), a function that returns counts and proportions of how many genes are present in a gene expression matrix for every input signature as well as possible missing genes.

Finally, the package supports the future framework to parallelize and speed-up computations either on a local machine or a computer cluster (Bengtsson, 2021).

More details about the usage of hacksig are reported in the package vignette, either on CRAN or running vignette(‘hacksig’) in R.

## 3 Conclusions and future perspectives

The R package hacksig offers a tidy and unified framework aimed at simplifying the computation of gene signature scores following both the original methods or three single sample alternatives. We acknowledge that our implementations of enrichment methods using ranks (i.e. ssGSEA and singscore) are slower than those in the GSVA and singscore packages (see Supplementary Material). Parallelization through the future R package is supported and can definitely decrease computation time. Nonetheless, the code for some functions might be rewritten in order to improve performance even more. More features are planned to be added, and we want to encourage future users of the package to open an issue on GitHub for every signature or method they would wish to be implemented.

## Supplementary Material

btac161_Supplementary_Data
