# Supplementary material for: hacksig: a unified and tidy R framework to easily compute gene expression signature scores
Source: Bioinformatics. 2022 Mar 18;38(10):2940–2. doi: 10.1093/bioinformatics/btac161 (PMC9113261; doi:10.1093/bioinformatics/btac161)
Supplement: btac161_Supplementary_Data [file btac161_supplementary_data.pdf]

hacksig: a unified and tidy R framework to easily compute gene expression signature scores

Supplementary materials

Carenzo, A., Pistore, F., Serafini, M.S., Lenoci, D., Licata A.G., De Cecco, L.

2022-02-24

Contents

Package design motivations **1**

Implemented signatures **2**

Comparison **2**

Performance 4

Output 4

Reproducible code **5**

Session info 9

References **12**

Package design motivations

Main motivations which lead us to rewrite from scratch the **three** single sample methods implementations (i.e. **combined z-score**, **single sample GSEA** and **singscore**) in `hacksig` are the following:

1. to keep as low as possible the number of package dependencies, favoring a `tidyverse` integration (Wickham et al. 2019);
2. to implement additional options for the single sample GSEA procedure (not present in the `GSVA` package), such as rank normalization as in the GenePattern module (see github.com/GSEA-MSigDB/ssGSEA-gpmodule and the help page of the `hack_sig()` function) and a sample-wise score normalization in $[0, 1]$ (see `sample_norm = "separate"` in `hack_sig()`);
3. to create a unified and tidy interface for these methods.

An important consequence from point 1 is that common Bioconductor classes, such as `SummarizedExperiment`, `ExpressionSet` or `GeneSetCollection`, are not supported (at the moment) (Huber et al. 2015). Hence, inputs to `hacksig` functions are simple base R objects: matrices or data frames for expression data sets and lists for gene sets. Nonetheless, if a consistent number of `hacksig` users will require a tighter integration with the Bioconductor ecosystem, we will surely consider this for future developments of the package.

Implemented signatures

Table S1 lists all the 23 cancer transcriptomics signatures implemented in `hacksig` as of February 2022. The *Primary category* column shows that most (17/23) of the signatures are immune-related. The *Type of tumor* column shows for which types of cancer a signature was developed.

In order to give a unique name to each signature, we adopted the following convention:

- **first author** name of the original publication;
- **year** of publication;
- underscore followed by a **keyword**.

Exceptions are signatures for the CINSARC, ESTIMATE and Immunophenoscore methods (Chibon et al. 2010; Yoshihara et al. 2013; Charoentong et al. 2017).

All functions in `hacksig` get gene-level information (e.g. gene symbols, model weights) about the implemented signatures from a hidden object called `signatures_data`:

```
hacksig:::signatures_data
```

```
## # A tibble: 878 x 8
##    signature_id  signature_keywords       gene_symbol gene_entrez_id gene_weight
##    <chr>         <chr>                    <chr>       <chr>                <dbl>
##  1 muro2016_ifng muro2016_ifng|ifng|inte~ CXCL9       4283                NA
##  2 muro2016_ifng muro2016_ifng|ifng|inte~ CXCL10      3627                NA
##  3 muro2016_ifng muro2016_ifng|ifng|inte~ IDO1        3620                NA
##  4 muro2016_ifng muro2016_ifng|ifng|inte~ IFNG        3458                NA
##  5 muro2016_ifng muro2016_ifng|ifng|inte~ HLA-DRA     3122                NA
##  6 muro2016_ifng muro2016_ifng|ifng|inte~ STAT1       6772                NA
##  7 fang2021_irgs fang2021_irgs|immune     DEFB1       1672                -0.319
##  8 fang2021_irgs fang2021_irgs|immune     EDNRB       1910                -0.418
##  9 fang2021_irgs fang2021_irgs|immune     ADM         133                  0.245
## 10 fang2021_irgs fang2021_irgs|immune     BTC         685                  0.710
## # ... with 868 more rows, and 3 more variables: signature_method <chr>,
## #   publication_doi <chr>, description <chr>
```

This object contains also the **keywords** used to obtain enrichment scores just for particular types of signatures (e.g. `signatures = "immune"` in `hack_sig()`) and the score computation **method** used in the original publication (e.g. average gene expression, weighted sum between gene expression values and model coefficients).

The `signatures_data` R object will be updated whenever new signatures will be added to `hacksig`.

Comparison

Now, we will compare single sample methods implemented in our package with those in `GSVA` and `singscore` (Hänzelmann et al. 2013; Foroutan et al. 2018). We will show differences in performance (i.e. computation time) and type of output by considering a toy gene expression matrix (randomly generated gene expression values for 20000 genes and 20 samples) included in `hacksig` and the 50 Hallmark gene sets (Liberzon et al. 2015).

Table S1: Cancer transcriptomics gene signatures implemented in hacksig.

Signature ID	Primary category	Typer of tumor	Description	Reference
estimate__immune	Immune	Solid cancers	Immune-related genes based on ESTIMATE (Estimation of STromal and Immune cells in MAlignant Tumours using Expression data).	Yoshihara et al. (2013)
estimate__stromal	Immune	Solid cancers	Stroma-related genes based on ESTIMATE (Estimation of STromal and Immune cells in MAlignant Tumours using Expression data).	Yoshihara et al. (2013)
rooney2015__cyt	Immune	Solid cancers	Genes giving a measure of immune cytolytic activity in tumors.	Rooney et al. (2015)
ips__cp	Immune	Solid cancers	Co-inhibitory and co-stimulatory biomarkers based on Immunophenoscore.	Charoentong et al. (2017)
ips__ec	Immune	Solid cancers	Infiltration of activated CD8+/CD4+ T cells and Tem CD8+/CD4+ cells based on Immunophenoscore.	Charoentong et al. (2017)
ips__mhc	Immune	Solid cancers	MHC class I/II and non-classical biomarkers based on Immunophenoscore.	Charoentong et al. (2017)
ips__sc	Immune	Solid cancers	Infiltration of immunosuppressive cells (Tregs and MDSCs) based on Immunophenoscore.	Charoentong et al. (2017)
muro2016__ifng	Immune	Melanoma	Interferon gamma-related genes found to be significantly associated with clinical response to pembrolizumab in patients with melanoma.	Muro et al. (2016)
ayers2017__immexp	Immune	Solid cancers	Immune expanded gene signature predicting response to pembrolizumab in multiple cancer types.	Ayers et al. (2017)
bai2019__immune	Immune	HNSCC-oral cavity	Immune/inflammatory-related prognostic genes.	Bai et al. (2019)
she2020__irgs	Immune	HNSCC	Prognostic signature of immune-related genes.	She et al. (2020)
liu2020__immune	Immune	HNSCC	Immune-related genes associated to overall survival.	Liu et al. (2020)
liu2021__mgs	Immune	HNSCC	Prognostic signature of myeloid-related genes.	Liu et al. (2021)
li2021__irgs	Immune	Thyroid cancer	Immune-related genes associated to dedifferentiation and immune exhaustion.	Li et al. (2021)
qiang2021__irgs	Immune	HNSCC	Prognostic signature of immune-related genes.	Qiang et al. (2021)
fang2021__irgs	Immune	HNSCC	Immune-related genes for survival prediction.	Fang et al. (2021)
eschrich2009__rsi	Prognostic/Predictive	Solid cancers	Genes aimed at predicting radiosensitivity. A higher index correspond to a more radioresistant patient.	Eschrich et al. (2009)
cinsarc	Prognostic/Predictive	Sarcoma	Signature (Complexity INdex in SARComas) aimed at identifying high-risk soft-tissue sarcomas and predicting metastasis outcome.	Chibon et al. (2010)
eustace2013__hypoxia	Prognostic/Predictive	HNSCC-larynx	Hypoxia-related genes aimed at predicting benefit from hypoxia-modifying treatment.	Eustace et al. (2013)
lohavanichbutr2013__hpvneg	Prognostic/Predictive	HNSCC-oral cavity	Signature prognostic of HPV-negative oral squamous cell carcinoma.	Lohavanichbutr et al. (2013)
dececco2014__int172	Prognostic/Predictive	HNSCC	Signature aimed at stratifying HNSCC patients in high/low risk of relapse.	De Cecco et al. (2014)
wu2020__metabolic	Prognostic/Predictive	HNSCC	Metabolic prognostic signature.	Wu et al. (2020)
hu2021__derbp	Prognostic/Predictive	HNSCC	Prognostic signature of genes related to RNA-binding proteins.	Hu et al. (2021)

Performance

Every benchmark will be run on a single core even if all three packages support parallel computation. The `bench` package (Hester and Vaughan 2021) will be used to run the functions 50 times each and compare the results for the *combined z-score*, *raw single sample GSEA* (ssGSEA) and *undirected singscore* methods. Benchmark results for the *normalized ssGSEA* and *up-regulated singscore* procedures will not be shown, being these similar in performance to the raw ssGSEA and undirected singscore respectively.

From Figure S1, the ssGSEA and singscore implementations in the `GSVA` and `singscore` packages respectively appear to be undoubtedly faster than those in `hacksig`.

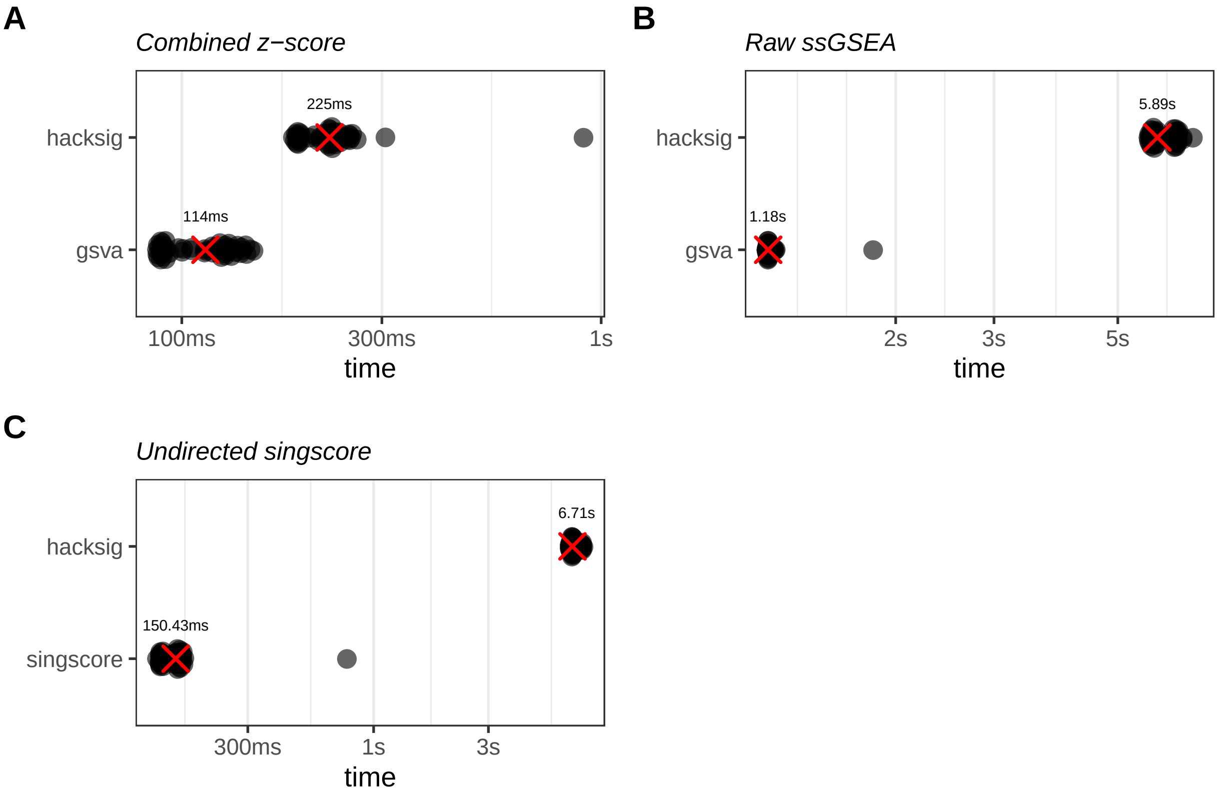

Figure S1: Benchmark times for hacksig versus GSVA/singscore implementations of combined z−score (A), raw ssGSEA (B) and undirected singscore (C). Red crosses show median times.

Output

Every dot in Figure S2 represents an enrichment score for the 50 Hallmark gene sets in 20 samples, computed both with `hacksig` and `GSVA`/`singscore`. Dots lay on the $y = x$ line and hence output from the `hack_sig()` function is equal to the corresponding `GSVA` and `singscore` counterparts.

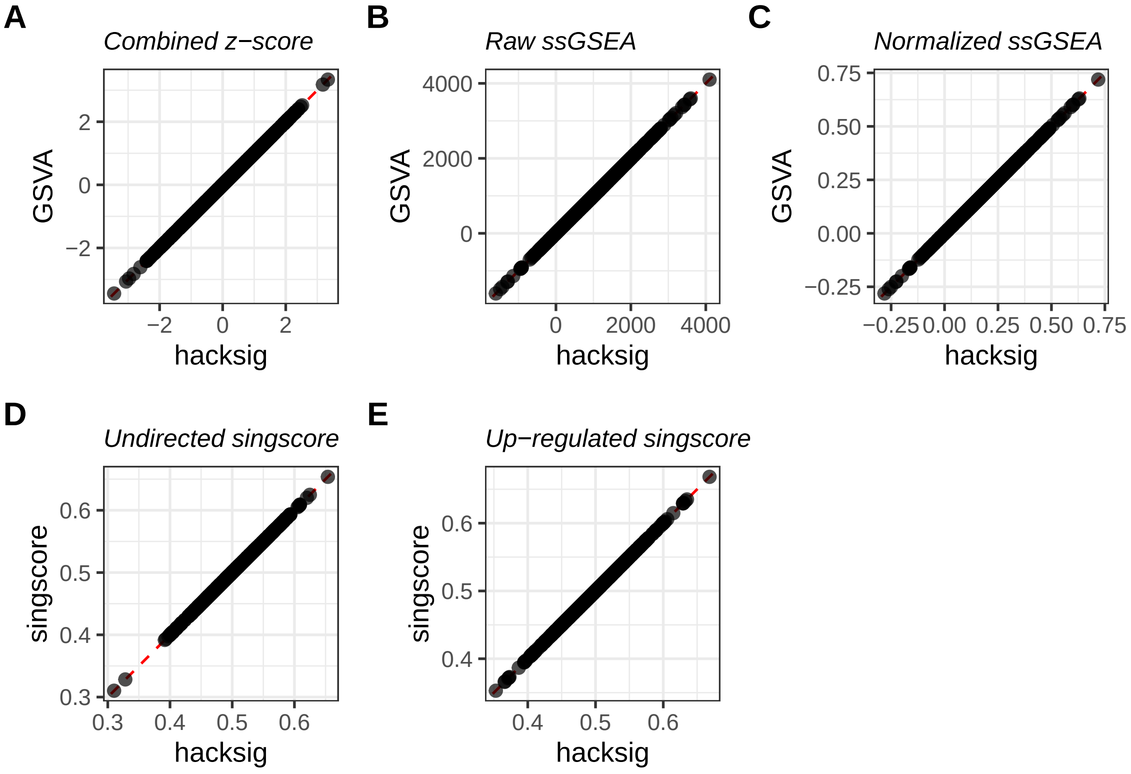

Figure S2: Enrichment scores comparison between hacksig and GSVA/singscore. Each panel shows enrichment scores computed with hacksig (x−axis) and GSVA or singscore (y−axis) for 5 different procedures: combined z−score (A), raw ssGSEA (B), normalized ssGSEA (C), undirected singscore (D), up−regulated singscore (E).

Reproducible code

This supplementary document was produced with `rmarkdown` (Xie et al. 2018). It is possible to reproduce output and figures above by running the R code reported in this section.

```
library(hacksig)
library(GSVA)
library(singscore)
library(dplyr)
library(ggplot2)
library(patchwork)

theme_set(theme_bw())
theme_update(panel.grid.major.y = element_blank())

# Store the Hallmark gene sets as a list of gene symbols
hallmark_list <- msigdbr::msigdbr(species = "Homo sapiens", category = "H") %>%
    distinct(gs_name, gene_symbol) %>%
    tidyr::nest(genes = c(gene_symbol)) %>%
    mutate(genes = purrr::map(genes,
                              purrr::compose(purrr::as_vector, unname))) %>%
    tibble::deframe()

# Benchmarks of hacksig versus GSVA/singscore
```

```r
res_zscore <- bench::mark(
    hacksig = hack_sig(expr_data = test_expr,
                       signatures = hallmark_list,
                       method = "zscore"),
    gsva = gsva(expr = test_expr, gset.idx.list = hallmark_list,
                method = "zscore", verbose = FALSE),
    iterations = 50, filter_gc = TRUE, check = FALSE
)

res_ssgsea <- bench::mark(
    hacksig = hack_sig(expr_data = test_expr,
                       signatures = hallmark_list,
                       method = "ssgsea", sample_norm = "raw"),
    gsva = gsva(expr = test_expr, gset.idx.list = hallmark_list,
                method = "ssgsea", ssgsea.norm = FALSE,
                verbose = FALSE),
    iterations = 50, filter_gc = TRUE, check = FALSE
)

ranked_expr <- rankGenes(test_expr, tiesMethod = "average")

hallmark_gsc <- purrr::pmap(
            list(hallmark_list,
                 setName = as.list(names(hallmark_list))),
            GSEABase::GeneSet
)

res_singscore <- bench::mark(
    hacksig = hack_sig(expr_data = test_expr,
                       signatures = hallmark_list,
                       method = "singscore", direction = "none"),
    singscore = multiScore(rankData = ranked_expr,
                           upSetColc = hallmark_gsc,
                           knownDirection = FALSE),
    iterations = 50, filter_gc = TRUE, check = FALSE
)

# Auxiliary function to plot benchmark results
plot_bench <- function(results, title, rev = FALSE) {
    p <- autoplot(results, color = "black",
             type = "beeswarm",
             alpha = 0.6, size = 3, width = 0.1) +
        stat_summary(fun = median,
                     color = "red", shape = 4, size = 1) +
        geom_text(aes(expression, time, label = median),
                  data = results %>%
                      mutate(time = gsub("m|s", "", median) %>%
                                 as.numeric() %>%
                                 ifelse(. > 100, . / 1000, .),
                             expression = as.character(expression),
                             median = as.character(median)),
                  size = 2,
                  nudge_x = 0.3, nudge_y = 0,
```

```
                   inherit.aes = FALSE) +
        labs(x = NULL, title = title)
    if (rev == TRUE) {
        p <- p + scale_x_discrete(limits = c("singscore", "hacksig"))
    }
    p
}

# Figure S1
fig1a <- plot_bench(res_zscore, "Combined z-score")
fig1b <- plot_bench(res_ssgsea, "Raw ssGSEA")
fig1c <- plot_bench(res_singscore, "Undirected singscore", TRUE)

fig1a + fig1b + fig1c +
    plot_layout(ncol = 2) +
    plot_annotation(tag_levels = "A",
                    caption = paste("Figure S1: Benchmark times for hacksig versus GSVA/singscore implem
                                    "raw ssGSEA (B) and undirected singscore (C). Red crosses show media
                                    sep = "\n")) &
    theme(plot.title = element_text(size = 10, face = "italic"),
          plot.tag = element_text(face = "bold"))

# Compute scores with different methods in hacksig and GSVA/singscore
## Combined z-score
z_hacksig <- hack_sig(expr_data = test_expr, signatures = hallmark_list,
                      method = "zscore") %>%
    tidyr::pivot_longer(-sample_id,
                        names_to = "sig", values_to = "hacksig_score")

z_gsva <- gsva(expr = test_expr, gset.idx.list = hallmark_list,
               method = "zscore", verbose = FALSE) %>%
    t() %>%
    tibble::as_tibble(rownames = "sample_id") %>%
    tidyr::pivot_longer(-sample_id,
                        names_to = "sig", values_to = "other_score")
## Raw ssGSEA
ssgraw_hacksig <- hack_sig(expr_data = test_expr, signatures = hallmark_list,
                           method = "ssgsea", sample_norm = "raw") %>%
    tidyr::pivot_longer(-sample_id,
                        names_to = "sig", values_to = "hacksig_score")

ssgraw_gsva <- gsva(expr = test_expr, gset.idx.list = hallmark_list,
                    method = "ssgsea", ssgsea.norm = FALSE,
                    verbose = FALSE) %>%
    t() %>%
    tibble::as_tibble(rownames = "sample_id") %>%
    tidyr::pivot_longer(-sample_id,
                        names_to = "sig", values_to = "other_score")
## Normalized ssGSEA
ssgnorm_hacksig <- hack_sig(expr_data = test_expr, signatures = hallmark_list,
                            method = "ssgsea", sample_norm = "all") %>%
    tidyr::pivot_longer(-sample_id,
                        names_to = "sig", values_to = "hacksig_score")
```

```r
ssgnorm_gsva <- gsva(expr = test_expr, gset.idx.list = hallmark_list,
                     method = "ssgsea", ssgsea.norm = TRUE,
                     verbose = FALSE) %>%
    t() %>%
    tibble::as_tibble(rownames = "sample_id") %>%
    tidyr::pivot_longer(-sample_id,
                        names_to = "sig", values_to = "other_score")
## Undirected singscore
singun_hacksig <- hack_sig(expr_data = test_expr, signatures = hallmark_list,
                           method = "singscore", direction = "none") %>%
    tidyr::pivot_longer(-sample_id,
                        names_to = "sig", values_to = "hacksig_score")

singun_singscore <- multiScore(rankData = ranked_expr,
                               upSetColc = hallmark_gsc,
                               knownDirection = FALSE) %>%
    purrr::pluck("Scores") %>%
    t() %>%
    tibble::as_tibble(rownames = "sample_id") %>%
    tidyr::pivot_longer(-sample_id,
                        names_to = "sig", values_to = "other_score")
## Up-regulated singscore
singup_hacksig <- hack_sig(expr_data = test_expr, signatures = hallmark_list,
                           method = "singscore", direction = "up") %>%
    tidyr::pivot_longer(-sample_id,
                        names_to = "sig", values_to = "hacksig_score")

singup_singscore <- multiScore(rankData = ranked_expr,
                               upSetColc = hallmark_gsc,
                               knownDirection = TRUE, centerScore = FALSE) %>%
    purrr::pluck("Scores") %>%
    t() %>%
    tibble::as_tibble(rownames = "sample_id") %>%
    tidyr::pivot_longer(-sample_id,
                        names_to = "sig", values_to = "other_score")

theme_set(theme_bw())
theme_update(plot.caption = element_text(hjust = 0))

# Auxiliary function to plot and compare output from hacksig and GSVA/singscore
compare_pkgs <- function(res_hacksig, res_other, y_label, title) {
    res_hacksig %>%
        left_join(res_other, by = c("sample_id", "sig")) %>%
        ggplot(aes(hacksig_score, other_score)) +
        geom_abline(intercept = 0, slope = 1, linetype = 2, color = "red") +
        geom_point(alpha = 0.7, size = 2) +
        coord_equal() +
        labs(x = "hacksig", y = y_label, title = title)
}

# Figure S2
fig2a <- compare_pkgs(z_hacksig, z_gsva, "GSVA", "Combined z-score")
fig2b <- compare_pkgs(ssgraw_hacksig, ssgraw_gsva, "GSVA", "Raw ssGSEA")
```

```r
fig2c <- compare_pkgs(ssgnorm_hacksig, ssgnorm_gsva, "GSVA",
                      "Normalized ssGSEA")
fig2d <- compare_pkgs(singun_hacksig, singun_singscore, "singscore",
                      "Undirected singscore")
fig2e <- compare_pkgs(singup_hacksig, singup_singscore, "singscore",
                      "Up-regulated singscore")

fig2a + fig2b + fig2c +
    fig2d + fig2e +
    plot_layout(ncol = 3) +
    plot_annotation(tag_levels = "A",
                    caption = paste("Figure S2: Enrichment scores comparison between hacksig and GSVA/si
                                    "scores computed with hacksig (x-axis) and GSVA or singscore (y-axi
                                    "z-score (A), raw ssGSEA (B), normalized ssGSEA (C), undirected sin
                                    sep = "\n")) &
    theme(plot.title = element_text(size = 10, face = "italic"),
          plot.tag = element_text(face = "bold"))
```

Session info

```
## - Session info ---------------------------------------------------------------
##  setting  value
##  version  R version 4.1.2 (2021-11-01)
##  os       Ubuntu 20.04.4 LTS
##  system   x86_64, linux-gnu
##  ui       X11
##  language (EN)
##  collate  en_US.UTF-8
##  ctype    en_US.UTF-8
##  tz       Europe/Rome
##  date     2022-02-24
##  pandoc   2.17.1.1 @ /usr/lib/rstudio/bin/quarto/bin/ (via rmarkdown)
##
## - Packages -------------------------------------------------------------------
##  package              * version  date (UTC) lib source
##  annotate               1.72.0   2021-10-26 [1] Bioconductor
##  AnnotationDbi          1.56.2   2021-11-09 [1] Bioconductor
##  assertthat             0.2.1    2019-03-21 [1] CRAN (R 4.1.2)
##  babelgene              21.4     2021-04-26 [1] CRAN (R 4.1.2)
##  beachmat               2.10.0   2021-10-26 [1] Bioconductor
##  beeswarm               0.4.0    2021-06-01 [1] CRAN (R 4.1.2)
##  bench                  1.1.2    2021-11-30 [1] CRAN (R 4.1.2)
##  Biobase                2.54.0   2021-10-26 [1] Bioconductor
##  BiocGenerics           0.40.0   2021-10-26 [1] Bioconductor
##  BiocParallel           1.28.3   2021-12-09 [1] Bioconductor
##  BiocSingular           1.10.0   2021-10-26 [1] Bioconductor
##  Biostrings             2.62.0   2021-10-26 [1] Bioconductor
##  bit                    4.0.4    2020-08-04 [1] CRAN (R 4.1.2)
##  bit64                  4.0.5    2020-08-30 [1] CRAN (R 4.1.2)
##  bitops                 1.0-7    2021-04-24 [1] CRAN (R 4.1.2)
##  blob                   1.2.2    2021-07-23 [1] CRAN (R 4.1.2)
##  cachem                 1.0.6    2021-08-19 [1] CRAN (R 4.1.2)
```

```
##  cellranger            1.1.0    2016-07-27 [1] CRAN (R 4.1.2)
##  cli                   3.2.0    2022-02-14 [1] CRAN (R 4.1.2)
##  codetools             0.2-18   2020-11-04 [4] CRAN (R 4.0.3)
##  colorspace            2.0-3    2022-02-21 [1] CRAN (R 4.1.2)
##  crayon                1.5.0    2022-02-14 [1] CRAN (R 4.1.2)
##  DBI                   1.1.2    2021-12-20 [1] CRAN (R 4.1.2)
##  DelayedArray          0.20.0   2021-10-26 [1] Bioconductor
##  DelayedMatrixStats    1.16.0   2021-10-26 [1] Bioconductor
##  digest                0.6.29   2021-12-01 [1] CRAN (R 4.1.2)
##  dplyr               * 1.0.8    2022-02-08 [1] CRAN (R 4.1.2)
##  edgeR                 3.36.0   2021-10-26 [1] Bioconductor
##  ellipsis              0.3.2    2021-04-29 [1] CRAN (R 4.1.2)
##  evaluate              0.15     2022-02-18 [1] CRAN (R 4.1.2)
##  fansi                 1.0.2    2022-01-14 [1] CRAN (R 4.1.2)
##  farver                2.1.0    2021-02-28 [1] CRAN (R 4.1.2)
##  fastmap               1.1.0    2021-01-25 [1] CRAN (R 4.1.2)
##  future                1.24.0   2022-02-19 [1] CRAN (R 4.1.2)
##  future.apply          1.8.1    2021-08-10 [1] CRAN (R 4.1.2)
##  generics              0.1.2    2022-01-31 [1] CRAN (R 4.1.2)
##  GenomeInfoDb          1.30.1   2022-01-30 [1] Bioconductor
##  GenomeInfoDbData      1.2.7    2021-12-06 [1] Bioconductor
##  GenomicRanges         1.46.1   2021-11-18 [1] Bioconductor
##  ggbeeswarm            0.6.0    2017-08-07 [1] CRAN (R 4.1.2)
##  ggplot2             * 3.3.5    2021-06-25 [1] CRAN (R 4.1.2)
##  globals               0.14.0   2020-11-22 [1] CRAN (R 4.1.2)
##  glue                  1.6.1    2022-01-22 [1] CRAN (R 4.1.2)
##  graph                 1.72.0   2021-10-26 [1] Bioconductor
##  GSEABase              1.56.0   2021-10-26 [1] Bioconductor
##  GSVA                * 1.42.0   2021-10-26 [1] Bioconductor
##  gtable                0.3.0    2019-03-25 [1] CRAN (R 4.1.2)
##  hacksig             * 0.1.2    2022-02-17 [1] CRAN (R 4.1.2)
##  HDF5Array             1.22.1   2021-11-14 [1] Bioconductor
##  htmltools             0.5.2    2021-08-25 [1] CRAN (R 4.1.2)
##  httr                  1.4.2    2020-07-20 [1] CRAN (R 4.1.2)
##  IRanges               2.28.0   2021-10-26 [1] Bioconductor
##  irlba                 2.3.5    2021-12-06 [1] CRAN (R 4.1.2)
##  kableExtra            1.3.4    2021-02-20 [1] CRAN (R 4.1.2)
##  KEGGREST              1.34.0   2021-10-26 [1] Bioconductor
##  knitr                 1.37     2021-12-16 [1] CRAN (R 4.1.2)
##  labeling              0.4.2    2020-10-20 [1] CRAN (R 4.1.2)
##  lattice               0.20-45  2021-09-22 [4] CRAN (R 4.1.1)
##  lifecycle             1.0.1    2021-09-24 [1] CRAN (R 4.1.2)
##  limma                 3.50.0   2021-10-26 [1] Bioconductor
##  listenv               0.8.0    2019-12-05 [1] CRAN (R 4.1.2)
##  locfit                1.5-9.4  2020-03-25 [1] CRAN (R 4.1.2)
##  magrittr              2.0.2    2022-01-26 [1] CRAN (R 4.1.2)
##  Matrix                1.4-0    2021-12-08 [4] CRAN (R 4.1.2)
##  MatrixGenerics        1.6.0    2021-10-26 [1] Bioconductor
##  matrixStats           0.61.0   2021-09-17 [1] CRAN (R 4.1.2)
##  memoise               2.0.1    2021-11-26 [1] CRAN (R 4.1.2)
##  msigdbr               7.4.1    2021-05-05 [1] CRAN (R 4.1.2)
##  munsell               0.5.0    2018-06-12 [1] CRAN (R 4.1.2)
##  parallelly            1.30.0   2021-12-17 [1] CRAN (R 4.1.2)
##  patchwork           * 1.1.1    2020-12-17 [1] CRAN (R 4.1.2)
```

```
##  pillar                 1.7.0    2022-02-01 [1] CRAN (R 4.1.2)
##  pkgconfig              2.0.3    2019-09-22 [1] CRAN (R 4.1.2)
##  plyr                   1.8.6    2020-03-03 [1] CRAN (R 4.1.2)
##  png                    0.1-7    2013-12-03 [1] CRAN (R 4.1.2)
##  profmem                0.6.0    2020-12-13 [1] CRAN (R 4.1.2)
##  purrr                  0.3.4    2020-04-17 [1] CRAN (R 4.1.2)
##  R6                     2.5.1    2021-08-19 [1] CRAN (R 4.1.2)
##  Rcpp                   1.0.8    2022-01-13 [1] CRAN (R 4.1.2)
##  RCurl                  1.98-1.6 2022-02-08 [1] CRAN (R 4.1.2)
##  readxl                 1.3.1    2019-03-13 [1] CRAN (R 4.1.2)
##  reshape2               1.4.4    2020-04-09 [1] CRAN (R 4.1.2)
##  rhdf5                  2.38.0   2021-10-26 [1] Bioconductor
##  rhdf5filters           1.6.0    2021-10-26 [1] Bioconductor
##  Rhdf5lib               1.16.0   2021-10-26 [1] Bioconductor
##  rlang                  1.0.1    2022-02-03 [1] CRAN (R 4.1.2)
##  rmarkdown              2.11     2021-09-14 [1] CRAN (R 4.1.2)
##  RSQLite                2.2.10   2022-02-17 [1] CRAN (R 4.1.2)
##  rstudioapi             0.13     2020-11-12 [1] CRAN (R 4.1.2)
##  rsvd                   1.0.5    2021-04-16 [1] CRAN (R 4.1.2)
##  rvest                  1.0.2    2021-10-16 [1] CRAN (R 4.1.2)
##  S4Vectors              0.32.3   2021-11-21 [1] Bioconductor
##  ScaledMatrix           1.2.0    2021-10-26 [1] Bioconductor
##  scales                 1.1.1    2020-05-11 [1] CRAN (R 4.1.2)
##  sessioninfo            1.2.2    2021-12-06 [1] CRAN (R 4.1.2)
##  SingleCellExperiment   1.16.0   2021-10-26 [1] Bioconductor
##  singscore            * 1.14.0   2021-10-26 [1] Bioconductor
##  sparseMatrixStats      1.6.0    2021-10-26 [1] Bioconductor
##  stringi                1.7.6    2021-11-29 [1] CRAN (R 4.1.2)
##  stringr                1.4.0    2019-02-10 [1] CRAN (R 4.1.2)
##  SummarizedExperiment   1.24.0   2021-10-26 [1] Bioconductor
##  svglite                2.1.0    2022-02-03 [1] CRAN (R 4.1.2)
##  systemfonts            1.0.4    2022-02-11 [1] CRAN (R 4.1.2)
##  tibble                 3.1.6    2021-11-07 [1] CRAN (R 4.1.2)
##  tidyr                  1.2.0    2022-02-01 [1] CRAN (R 4.1.2)
##  tidyselect             1.1.2    2022-02-21 [1] CRAN (R 4.1.2)
##  utf8                   1.2.2    2021-07-24 [1] CRAN (R 4.1.2)
##  vctrs                  0.3.8    2021-04-29 [1] CRAN (R 4.1.2)
##  vipor                  0.4.5    2017-03-22 [1] CRAN (R 4.1.2)
##  viridisLite            0.4.0    2021-04-13 [1] CRAN (R 4.1.2)
##  webshot                0.5.2    2019-11-22 [1] CRAN (R 4.1.2)
##  withr                  2.4.3    2021-11-30 [1] CRAN (R 4.1.2)
##  xfun                   0.29     2021-12-14 [1] CRAN (R 4.1.2)
##  XML                    3.99-0.8 2021-09-17 [1] CRAN (R 4.1.2)
##  xml2                   1.3.3    2021-11-30 [1] CRAN (R 4.1.2)
##  xtable                 1.8-4    2019-04-21 [1] CRAN (R 4.1.2)
##  XVector                0.34.0   2021-10-26 [1] Bioconductor
##  yaml                   2.3.5    2022-02-21 [1] CRAN (R 4.1.2)
##  zlibbioc               1.40.0   2021-10-26 [1] Bioconductor
##
##  [1] /home/andrea/R/x86_64-pc-linux-gnu-library/4.1
##  [2] /usr/local/lib/R/site-library
##  [3] /usr/lib/R/site-library
##  [4] /usr/lib/R/library
##
```

References

Ayers M, Lunceford J, Nebozhyn M, et al (2017) IFN-γ–related mRNA profile predicts clinical response to PD-1 blockade. Journal of Clinical Investigation 127:2930–2940. https://doi.org/10.1172/jci91190

Bai S, Yan Y-B, Chen W, et al (2019) Bioinformatic analysis reveals an immune/inflammatory-related risk signature for oral cavity squamous cell carcinoma. Journal of Oncology 2019:1–10. https://doi.org/10.1155/2019/3865279

Charoentong P, Finotello F, Angelova M, et al (2017) Pan-cancer immunogenomic analyses reveal genotype-immunophenotype relationships and predictors of response to checkpoint blockade. Cell Rep 18:248–262. https://doi.org/10.1016/j.celrep.2016.12.019

Chibon F, Lagarde P, Salas S, et al (2010) Validated prediction of clinical outcome in sarcomas and multiple types of cancer on the basis of a gene expression signature related to genome complexity. Nat Med 16:781–787. https://doi.org/10.1038/nm.2174

De Cecco L, Bossi P, Locati L, et al (2014) Comprehensive gene expression meta-analysis of head and neck squamous cell carcinoma microarray data defines a robust survival predictor. Ann Oncol 25:1628–1635. https://doi.org/10.1093/annonc/mdu173

Eschrich SA, Pramana J, Zhang H, et al (2009) A gene expression model of intrinsic tumor radiosensitivity: Prediction of response and prognosis after chemoradiation. International Journal of Radiation Oncology Biology Physics 75:489–496. https://doi.org/10.1016/j.ijrobp.2009.06.014

Eustace A, Mani N, Span PN, et al (2013) A 26-gene hypoxia signature predicts benefit from hypoxia-modifying therapy in laryngeal cancer but not bladder cancer. Clinical Cancer Research 19:4879–4888. https://doi.org/10.1158/1078-0432.ccr-13-0542

Fang R, Iqbal M, Chen L, et al (2021) A novel comprehensive immune-related gene signature as a promising survival predictor for the patients with head and neck squamous cell carcinoma. Aging 13:11507–11527. https://doi.org/10.18632/aging.202842

Foroutan M, Bhuva DD, Lyu R, et al (2018) Single sample scoring of molecular phenotypes. BMC bioinformatics 19: https://doi.org/10.1186/s12859-018-2435-4

Hänzelmann S, Castelo R, Guinney J (2013) GSVA: Gene set variation analysis for microarray and RNA-seq data. BMC bioinformatics 14: https://doi.org/10.1186/1471-2105-14-7

Hester J, Vaughan D (2021) Bench: High precision timing of r expressions

Hu G, Jiang Q, Liu L, et al (2021) Integrated analysis of RNA-binding proteins associated with the prognosis and immunosuppression in squamous cell carcinoma of head and neck. Frontiers in Genetics 11: https://doi.org/10.3389/fgene.2020.571403

Huber W, Carey VJ, Gentleman R, et al (2015) Orchestrating high-throughput genomic analysis with bioconductor. Nature Methods 12:115–121. https://doi.org/10.1038/nmeth.3252

Li C-W, Shi X, Ma B, et al (2021) A 4 gene-based immune signature predicts dedifferentiation and immune exhaustion in thyroid cancer. The Journal of Clinical Endocrinology & Metabolism 106:e3208–e3220. https://doi.org/10.1210/clinem/dgab132

Liberzon A, Birger C, Thorvaldsdóttir H, et al (2015) The molecular signatures database hallmark gene set collection. Cell Syst 1:417–425. https://doi.org/10.1016/j.cels.2015.12.004

Liu X, Chen J, Lu W, et al (2020) Systematic profiling of immune risk model to predict survival and immunotherapy response in head and neck squamous cell carcinoma. Frontiers in Genetics 11: https://doi.org/10.3389/fgene.2020.576566

Liu Z, Zhang D, Liu C, et al (2021) Comprehensive analysis of myeloid signature genes in head and neck squamous cell carcinoma to predict the prognosis and immune infiltration. Frontiers in Immunology 12: https://doi.org/10.3389/fimmu.2021.659184

Lohavanichbutr P, Méndez E, Holsinger FC, et al (2013) A 13-gene signature prognostic of HPV-negative OSCC: Discovery and external validation. Clinical Cancer Research 19:1197–1203. https://doi.org/10.1158/1078-0432.ccr-12-2647

Muro K, Chung HC, Shankaran V, et al (2016) Pembrolizumab for patients with PD-L1-positive advanced gastric cancer (KEYNOTE-012): A multicentre, open-label, phase 1b trial. The Lancet Oncology 17:717–

726. https://doi.org/10.1016/s1470-2045(16)00175-3
Qiang W, Dai Y, Xing X, Sun X (2021) Identification and validation of a prognostic signature and combination drug therapy for immunotherapy of head and neck squamous cell carcinoma. Computational and Structural Biotechnology Journal 19:1263–1276. https://doi.org/10.1016/j.csbj.2021.01.046
Rooney MS, Shukla SA, Wu CJ, et al (2015) Molecular and genetic properties of tumors associated with local immune cytolytic activity. Cell 160:48–61. https://doi.org/10.1016/j.cell.2014.12.033
She Y, Kong X, Ge Y, et al (2020) Immune-related gene signature for predicting the prognosis of head and neck squamous cell carcinoma. Cancer Cell International 20: https://doi.org/10.1186/s12935-020-1104-7
Wickham H, Averick M, Bryan J, et al (2019) Welcome to the tidyverse. JOSS 4:1686. https://doi.org/10.21105/joss.01686
Wu Z-H, Tang Y, Zhou Y (2020) A metabolic gene signature to predict overall survival in head and neck squamous cell carcinoma. Mediators of Inflammation 2020:1–12. https://doi.org/10.1155/2020/6716908
Xie Y, Allaire JJ, Grolemund G (2018) R markdown: The definitive guide. Chapman; Hall/CRC, Boca Raton, Florida
Yoshihara K, Shahmoradgoli M, Martínez E, et al (2013) Inferring tumour purity and stromal and immune cell admixture from expression data. Nat Commun 4: https://doi.org/10.1038/ncomms3612
